# Autoreactive Peripheral Blood T Helper Cell Responses in Bullous Pemphigoid and Elderly Patients With Pruritic Disorders

**DOI:** 10.3389/fimmu.2021.569287

**Published:** 2021-03-25

**Authors:** Dario Didona, Luca Scarsella, Milad Fehresti, Farzan Solimani, Hazem A. Juratli, Manuel Göbel, Stefan Mühlenbein, Lily Holiangu, Josquin Pieper, Vera Korff, Thomas Schmidt, Cassian Sitaru, Rüdiger Eming, Michael Hertl, Robert Pollmann

**Affiliations:** ^1^ Department of Dermatology and Allergology, Philipps-Universität Marburg, Marburg, Germany; ^2^ Department of Dermatology, Venereology and Allergology, Charité-Universitätsmedizin Berlin, Berlin, Germany; ^3^ Department of Dermatology and Allergology, Universität Freiburg, Freiburg, Germany

**Keywords:** autoimmunity, pemphigoid, BP180, T cells, ELISpot assay, pruritus

## Abstract

Bullous pemphigoid (BP) is a prototypic autoimmune disorder of the elderly, characterized by serum IgG autoantibodies, namely anti-BP180 and anti-BP230, directed against components of the basal membrane zone that lead to sub-epidermal loss of adhesion. Pruritus may be indicative of a pre-clinical stage of BP, since a subset of these patients shows serum IgG autoantibodies against BP230 and/or BP180 while chronic pruritus is increasingly common in the elderly population and is associated with a variety of dermatoses. Clinical and experimental evidence further suggests that pruritus of the elderly may be linked to autoimmunity with loss of self-tolerance against cutaneous autoantigens. Thus, the objective of this study was to determine autoreactive T cell responses against BP180 in elderly patients in comparison to patients with BP. A total of 22 elderly patients with pruritic disorders, 34 patients with bullous or non-bullous BP and 34 age-matched healthy controls were included in this study. The level of anti-BP180 and anti-BP230 IgG serum autoantibodies, Bullous Pemphigoid Disease Area Index (BPDAI), and pruritus severity were assessed for all patients and controls. For characterization of the autoreactive T cell response, peripheral blood mononuclear cells were stimulated *ex vivo* with recombinant BP180 proteins (NH_2_- and COOH-terminal domains) and the frequencies of BP180-specific T cells producing interferon-γ, interleukin (IL)-5 or IL-17 were subsequently determined by ELISpot assay. Patients with BP showed a mixed Th1/Th2 response against BP180 while autoreactive Th1 cells were identified in a minor subset of elderly patients with pruritic disorders. Furthermore, our T cell characterization revealed that therapeutic application of topical clobetasol propionate ointment in BP patients significantly reduced peripheral blood BP180-specific T cells, along with clinically improved symptoms, strongly suggesting a systemic immunosuppressive effect of this treatment.

## Introduction

Bullous pemphigoid (BP) is the most common autoimmune sub-epidermal blistering skin disease, which usually affects people in the 6th to 8th decade of life and is clinically characterized by bullous and non-bullous skin lesions (1–3). Since an intense pruritus is also present in BP, it has been thought that pruritic disorders of the elderly may represent a prodromal stage of BP (4–6). Pruritus frequently occurs in the elderly population (7) with a worldwide prevalence between 7.3% and 41% (8–10). In Germany a prevalence of 22.0% has been reported (11). Immunological alterations, like immune senescence and increased loss of tolerance to self-antigens, can play a key role in the etiology of pruritus of the elderly (10, 12). Immune senescence leads to a reduction of T helper (Th)1 cell-driven immunity and a shift towards Th2 polarization, indicated by elevated serum antibody levels, peripheral eosinophilia, and/or tissue eosinophil infiltration (13) and a reduced activity of T regulatory (Treg) cells (14, 15). In addition, a reduction of CD8+ T cells has been reported in elderly patients (EP) with pruritic disorders, which further supports the concept of an age-dependent CD4+ Th2 polarization as a potential factor to the development of pruritus (4, 13, 16).

The most common clinical subtype of pemphigoid diseases is classical generalized BP, characterized by tense blisters on erythematous skin on the trunk and extremities (2, 17). Other rarer variants, such as localized BP, mucous membrane pemphigoid, anti-laminin-gamma-1 pemphigoid, and childhood BP, have been also described (18, 19). Furthermore, in the early stages of the disease, blisters may not be present, but eczematous or urticarial lesions can be observed (2, 20). In approximately 20% of BP patients, these skin features are the only signs of the disease, while skin blisters are not observed (21, 22). This clinical subtype is known as non-bullous pemphigoid (nBP) (23, 24). The immunological hallmark of BP is serum IgG autoantibodies against the hemidesmosomal components BP180 and BP230, which are critical for dermo-epidermal adhesion (25). BP180 is a 180-kDa transmembrane protein; its non-collagenous domain-16a (NC-16a) is recognized by the majority of BP patient sera (26). Autoreactive T cells against BP180 are essential drivers of the autoimmune response against BP180, inducing and amplifying the production of serum autoantibodies in BP. Autoreactive T cells, which predominantly target distinct epitopes of the BP180-NH2-terminus (including the immunodominant NC16A domain), while the COOH-terminus is recognized to a lesser extent, produce pro-inflammatory cytokines, such as interleukin (IL)-5, interferon-γ (IFN-γ), and presumably also IL-17 (27–30). Furthermore, it has been highlighted that Th17 cells may also play a role in the pathogenesis of BP, as IL-17 may induce the expression of proinflammatory cytokines such as tumor necrosis factor-α (TNF-α), IL-1b and IL-6 (31). In addition, IgG autoantibodies against BP230 have been detected in sera of EP with pruritic disorders despite the absence of tissue bound IgG at the basement membrane zone (BMZ) (4).

We here aimed to characterize autoreactive T cells against BP180 in peripheral blood of patients with classical generalized BP and nBP in comparison to elderly patients with pruritic disorders.

## Materials and Methods

### Patients

A total of 56 patients were enrolled in this study. This cohort consisted of three sub-groups: a) EP with pruritic disorders (n=22) b) patients with nBP (n=5) and c) patients with classical BP (n=29). The diagnosis of BP and nBP was based on the presence of linear IgG and/or C3 deposits along the dermo-epidermal junction by direct immunofluorescence (DIF) and circulating IgG anti-basement membrane IgG autoantibodies by indirect immunofluorescence (IIF) microscopy on human salt-split-skin or ELISA according to the latest European guidelines (32). EP with pruritic disorders were by definition negative by DIF despite the presence of serum IgG against BP180 and/or BP230 in individual patients. EP were affected by pruritus and pruriginous plaques and papules, without any underlying specific dermatosis. Patients with comorbidities, such as hypertension or hypertriglyceridemia, were only included if a long-term concomitant medication was taken to exclude any potential drug-related itching. Only emollients but no topical anti-inflammatory treatment was applied throughout the study. Any presence of impaired liver or kidney function was excluded. Patients with nBP were off treatment, while the group of BP patients consisted of 13 newly diagnosed naïve patients without any treatment and 16 patients treated with topical corticosteroids. The study also included 34 age-matched healthy controls (HC). All participants signed a written consent for inclusion into the study. The study protocol was reviewed and approved by the Ethics Committee of the Medical Faculty of Philipps University, Marburg (no. 136/11).

### Clinical Severity Assessment

The clinical activity was assessed for all patient groups by the Bullous Pemphigoid Disease Area Index (BPDAI). BPDAI is a validated index ideated to evaluate extent and type of BP lesions and has been largely used in experimental and clinical studies on BP (33, 34). It ranges from 0 to 360 and it is based on three different parameters: i) cutaneous blisters, ii) urticarial/erythematous lesions, and iii) mucosal blisters/erosions (35). The BPDAI score was also applied in EP in order to compare the clinical symptoms with BP patients. Pruritus severity within the last 24 hours was evaluated through a subjective scale from 0 to 10, where 0 represents no itching and 10 represents maximal itching.

### Immune Serological Analyses

Serum concentrations of anti-BP180 IgG and anti-BP230 IgG autoantibodies were determined by a commercial ELISA, i.e. anti-BP180-NC16A-4X and anti-BP230-CF ELISA kits (all Euroimmun, Lübeck, Germany) according to the manufacturer’s instructions.

### ELISpot Assay

Peripheral blood was collected in Citrate-Phosphate-Dextrose-Adenin (CPDA) monovettes and Peripheral Blood Mononuclear Cells (PBMC) were isolated using density gradient centrifugation. Isolated PBMC were cryopreserved in freezing medium (90% FCS and 10% DMSO) and stored in liquid nitrogen prior to ELISpot analysis. ELISpot analysis was performed as described in elsewhere (36, 37). Briefly, PBMC were thawed and cultured in RPMI + 10% pooled human serum and stimulated with 10 µg/ml recombinant BP180-NH2 (aa490-812) or BP180-COOH (aa1352-1465) protein produced in a baculovirus expression system (29, 38). After 3 days of culture, recombinant IL-2 (10 U/ml) and IL-7 (10 ng/ml) were added for expansion of activated BP180-specific T cells. After 7 days, stimulated cells were transferred to 96 well plates coated with anti-human IL-5, anti-IL-17, or anti-IFN-γ and restimulated for 18 hours with BP180-NH2 or BP180-COOH. 2 × 10^5^ cells (IL-5, IL-17) or 1 × 10^5^ (IFN-γ) cells were seeded per well. After assay development, membrane-bound spots were counted automatically with an ELISpot plate reader (A.EL.VIS, Hannover, Germany). For data analysis, spots of non-stimulated controls (mean) were subtracted from spots (mean) of cultures with antigen (all in duplicate).

### Statistical Analysis

Statistical analysis was performed using One Way ANOVA or Mann Whitney U test; a *p*-value < 0.05 was considered statistically significant. Association between continuous variables was assessed using Spearman’s rank correlation test values range from -1 (perfect negative linear correlation) to +1 (perfect positive linear relationship), a value of 0 indicates no linear correlation between two variables. Statistical analysis was performed using GraphPad Prism version 4.0.

## Results

### Detection of Serum Anti-BP180 and Anti-BP230 IgG in EP With Pruritic Disorders and BP Patients

**Figure 1:**
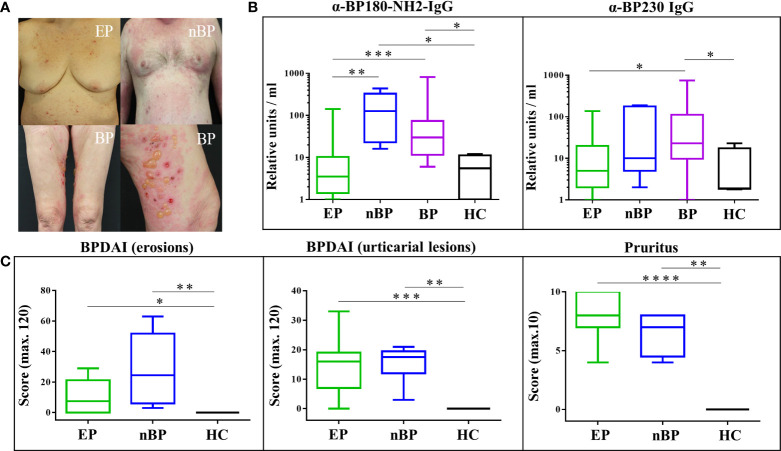
Clinical and immune serological profile of elderly patients (EP) with pruritic disorders, non-bullous pemphigoid (nBP), bullous pemphigoid (BP), and healthy controls (HC). **(A)** Clinical appearance of EP, nBP and BP. **(B)** Anti-BP180 and anti-BP230 serum IgG autoantibodies. **(C)** Bullous Pemphigoid Disease Area Index (BPDAI) and pruritus assessment (severity within the last 24 hours). Statistical analysis of the study groups was performed using Mann–Whitney U-test (*P < 0.05, **P < 0.01, ***P < 0.001, ****P < 0.0001).

The studied cohort of EP with pruritic disorders showed a rather heterogeneous clinical phenotype with pruriginous plaques and papules with some overlap to nBP, while patients with classical BP presented with tense blisters (**Figure 1A**). Serum levels of IgG autoantibodies against BP180 and BP230 were evaluated by ELISA and are shown in **Figure 1B** and **Supplementary Table 1**. Specifically, nBP and BP patients showed the highest serum concentrations of IgG autoantibodies. Compared to HC, anti-BP180-NH2 IgG serum concentrations were significantly increased in nBP patients (*p* = 0.0159) and BP patients (*p* = 0.0220), while anti-BP230 serum IgG was significantly increased only in BP patients (*p* = 0.0461). In contrast, EP with pruritic disorders showed anti-BP180-NH2- and anti-BP230-IgG serum concentrations that did not statistically differ from the HC group, even though individual EP with pruritic disorders showed serum IgG against BP180-NH2 (n=2; 75 and 142 RU/ml) and BP230 (n=4; 26-137 RU/ml). Skin erosions, urticarial plaques, and severity of pruritus were increased in EP with pruritic disorders and nBP patients compared to HC.

### ELISpot Assay With BP180-NH2 and BP180-COOH Recombinant Antigens

**Figure 2:**
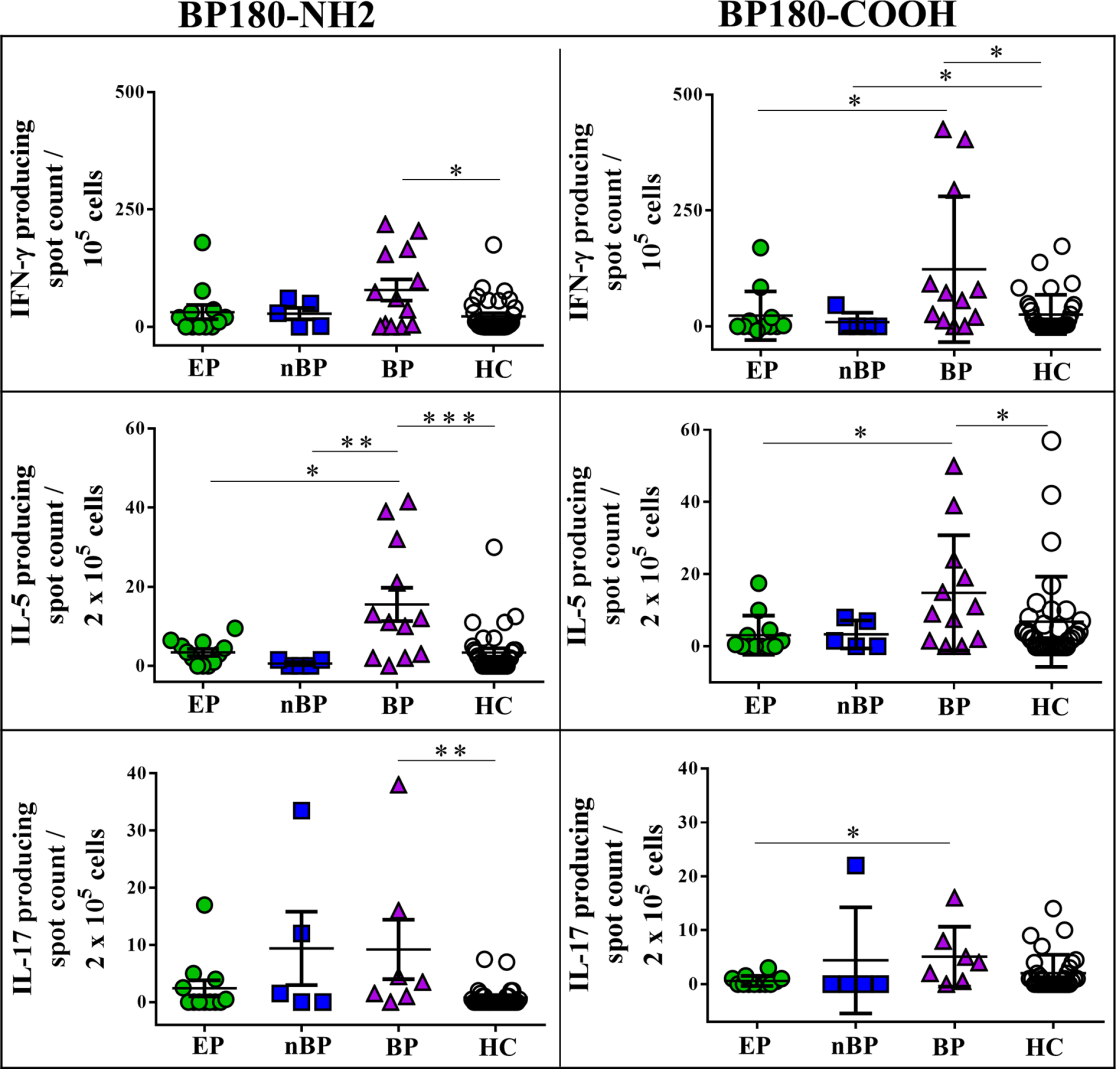
Peripheral blood T cell responses against bullous pemphigoid (BP) 180 in elderly patients with pruritic disorders (EP), non-bullous pemphigoid (nBP), and healthy controls (HC). Shown are cytokine secreting T cells, producing IFN-γ, IL-5 or IL-17 upon *ex vivo* stimulation with the immunodominant NC16a domain of BP180 (BP180-NH2) or the COOH terminus of BP180 (BP180-COOH). Differences between the groups were analyzed using Mann-Whitney U-test (*P < 0.05, **P < 0.01, ***P < 0.001).

To evaluate autoreactive T cell responses against BP180, the major autoantigen of BP, in all patient cohorts, we determined cytokine release of peripheral blood T cells upon *ex vivo* stimulation with BP180-NH2- and BP180-COOH-recombinant proteins using ELISpot assay (**Figure 2**, **Supplementary Table 2**). Autoreactive T cells producing IFN-γ, IL-5, and IL-17 were highly increased in BP patients as compared to HC. Overall, EP with pruritic disorders showed no significantly increased autoreactive T cells specific for BP180-NH2 and BP180-COOH subdomains. However, individual EP with pruritic disorders showed autoreactive T cells with a cytokine profile similar to BP patients (**Figure 2**). Moreover, the group of nBP patients showed a trend towards increased IL-17-producing T cell responses against BP180, which was not statistically significant in comparison to HC. Notably, Th1 cell responses against BP180-NH2 and BP180-COOH could also be observed in individual HC.

### Correlation Analysis of Peripheral Blood Autoreactive T Cell Responses Against BP180 in BP Patients and EP With Pruritic Disorders

**Figure 3:**
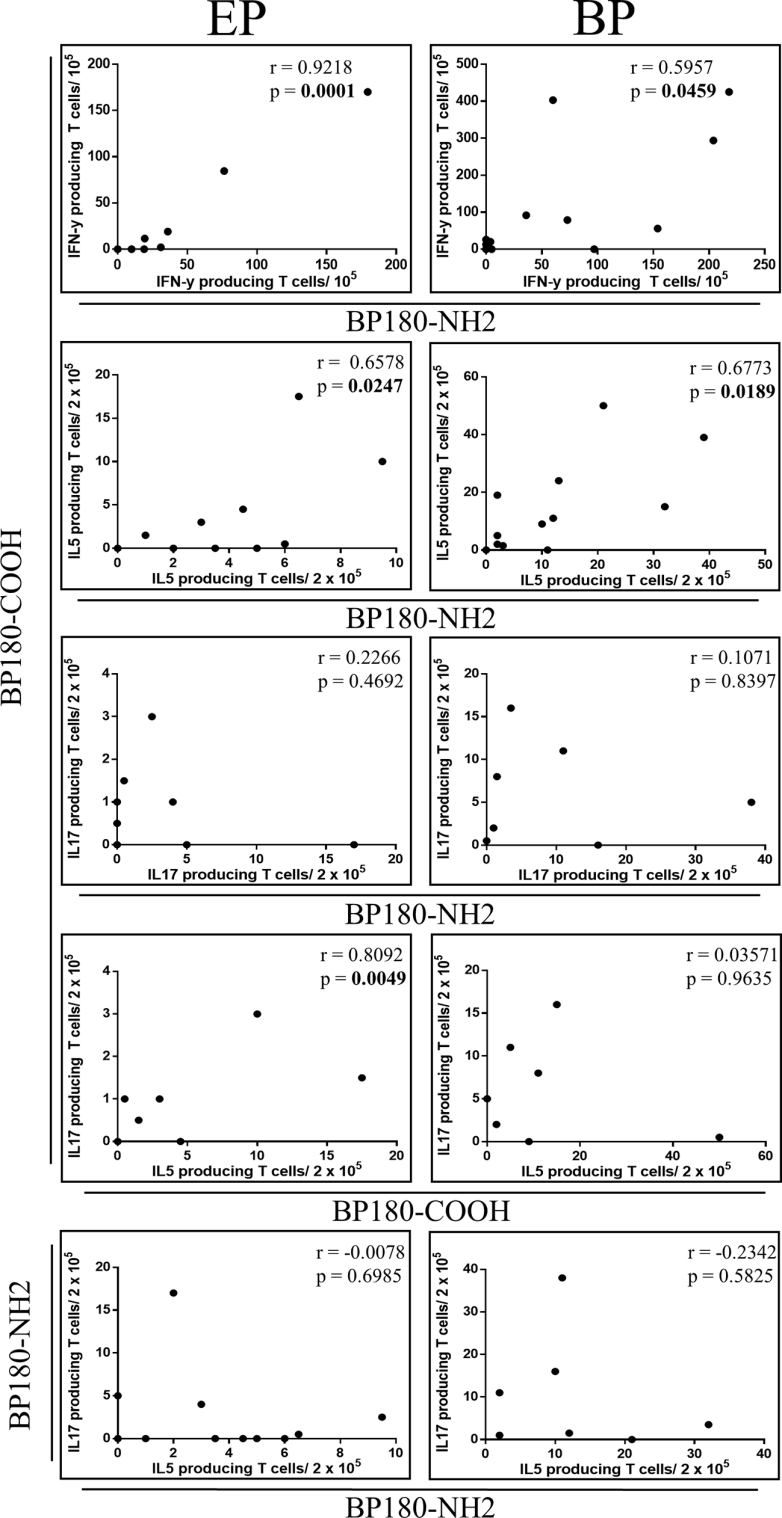
Relationship between pro- and anti-inflammatory autoreactive T cell responses against bullous pemphigoid (BP) 180 in elderly patients with pruritic disorders (EP). Plots showing correlations of anti-inflammatory and pro-inflammatory cytokine producing T cells specific for BP180-NH2 or BP180-COOH, respectively. From top down: relationship between IFN-γ producing T cells, IL-5 and IL-17, upon stimulation with BP180-COOH or BP180-NH2 between EP and BP patients. Correlation between IL-17-producing T cells and IL-5-producing T cells upon stimulation with BP180-COOH for EP and BP patients. Correlation between IL17-producing T cells and IL-5-producing T cells upon stimulation with BP180-NH2. Spearman’s correlation coefficients (r) are shown with significant p values (p <0.05) highlighted in bold.

Since we observed autoreactive T cell responses against BP180 in BP patients we evaluated whether this response was directed to a single or several BP180 subdomains. Accordingly, Spearman’s correlation analysis was performed with IFN-γ-, IL-5-, IL-17-producing autoreactive T cells reactive against BP180-NH2 or BP180-COOH respectively (**Figure 3**, **Supplementary Table 3**). Correlation analysis was compared with EP patients as they showed BP180-specific T cell responses in individual patients, although the frequency of BP180-specific T cells in EP with pruritic disorders was reduced compared to BP patients (**Figure 2**). Of note, a positive correlation of IFN-γ- and IL-5-producing T cells and BP180-NH2- or BP180-COOH was observed in both groups. However, a similar correlation for IL-17-producing T cells was not observed for EP with pruritic disorders and BP patients. Furthermore, only in EP with pruritic disorders, IL-17-secretion of autoreactive T cells against BP180-COOH correlated with IL-5 production, while this was not detected for the BP180-NH2 subdomain. These data suggest a common Th1 and Th2 response in patients with BP and to a lesser extent in individual EP patients.

### Effect of the Treatment With Topical Corticosteroid on Peripheral T Cell Responses Against BP180

**Figure 4:**
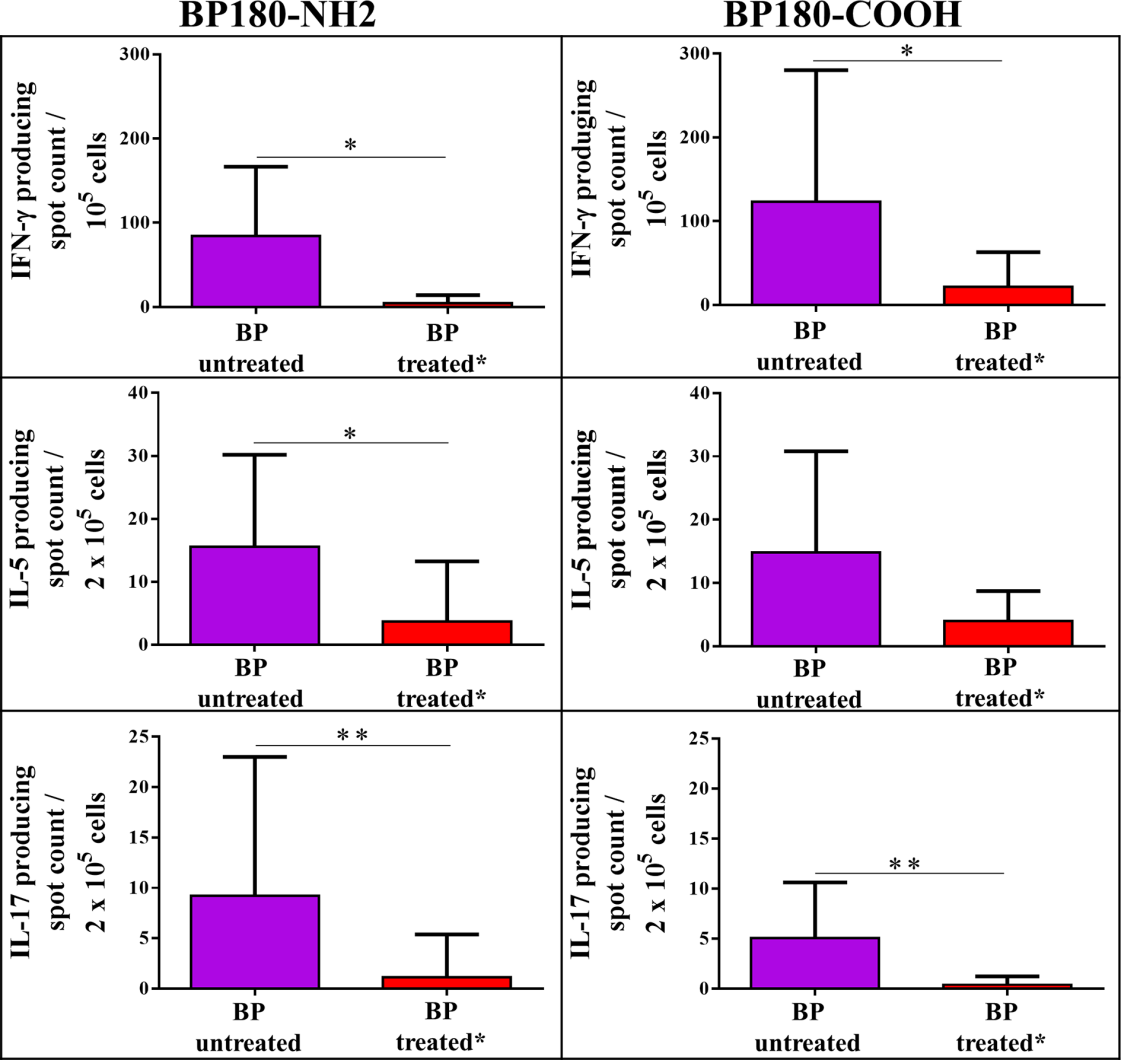
Impact of treatment with topical glucocorticoids (clobetasol propionate ointment) on peripheral blood T cell responses against bullous pemphigoid (BP) 180 in BP patients. Shown are T cells producing IFN-γ, IL-5 or IL-17 upon *ex vivo* stimulation with the immunodominant NH2-terminal NC16a domain of BP180 (BP180-NH2) or the COOH terminus of BP180 (BP180-COOH), respectively. BP patients with active disease were either untreated or had been treated topically with 0.05% clobetasol propionate ointment for at least 5 days. Differences between the groups were analyzed using Mann-Whitney U-test for non-parametric data (*p < 0.05, **p < 0.01).

A subgroup of BP patients (n=16) received treatment with clobetasol propionate ointment. This group led us to evaluate the effect of topically applied high potency glucocorticoids on autoreactive BP180-specific T cells in peripheral blood of BP patients (**Figure 4** **)**. Remarkably, topical treatment led to a massive reduction of IFN-γ-, IL-5-, and IL-17-producing autoreactive T cells reactive against the BP180-NH2 and BP180-COOH subdomains, indicating a systemic effect of the topical immunosuppression.

## Discussion

This is the first study evaluating T cell responses against BP180 in patients with classical BP and nBP in comparison to EP with pruritic disorders. IgG autoantibodies against BP180 and BP230 have already been described in a subset of EP with pruritic disorders (23, 39) which in contrast to classical BP do not show IgG- and/or C3-deposits at the dermal-epidermal BMZ (4). It is assumed that these individuals are prone to develop BP, as anti‐BP230 and/or anti‐BP180‐specific IgG serum autoantibodies may provide a link between pruritus and autoimmunity against the BMZ (23, 39). Here, we observed that only a minor subset of EP patients with pruritic disorders showed serum IgG autoantibodies against BP230 and BP180 (**Figure 1**). Along this line, only a few EP with pruritic disorders showed peripheral blood T cell responses against the NH2-terminus and/or the COOH-terminus of BP180 (**Figure 2**). These findings suggest that a small subset of EP with pruritic disorders shows autoimmune response against the skin-derived BP180 protein and may thus be at risk to developing BP. A long-term follow up of these patients would allow to further enlighten the potential link between autoreactivity against BP180 and the development of BP in EP which could not be performed in our initial cross-sectional pilot study.

Noteworthy, BP180-NH2-specific T cells from EP with pruritic disorders preferentially secreted the Th1-related cytokine IFN-γ, while autoreactive T cells from BP patients preferentially secreted both, Th1 (IFN-γ) and Th2 (IL-5)-related cytokines. This finding is in line with previous studies showing that BP patients preferentially mount a Th2 cell response against BP180, the major autoantigen of BP (27, 29). It has been reported in a recent study that following activation with the type 2 cytokine, IL-5, eosinophils induced separation in the skin along the dermal-epidermal junction of skin in the presence of BP autoantibodies (40). Increasing levels of IL-5 and IFN-γ in blister fluids of BP patients further point towards a specific role of type 1 and type 2 T cell-associated cytokines in the pathogenesis of BP (41).

In our study, we detected Th17 cell responses against BP180 in a few patients with BP and only to a minor extent in EP with pruritic disorders. The role of IL-17-producing T cells in BP has been partially elucidated (30, 42–46). Recently, it was shown that mRNA levels of IL-17 were upregulated in perilesional skin of BP patients (30, 46) and inhibition of IL-17 with anti-IL-17 antibodies prevents BP180 IgG-induced blister formation (30, 46). Overall, the activation of the Th17 pathway seems to support the development of inflammation by recruiting neutrophils and eosinophils, and inducing the production of pro-inflammatory cytokines and proteolytic enzymes (30, 47). However, the exact role of Th17 cells in BP has not yet been fully elucidated (43, 46). In our study, BP180-NH2- and BP180-COOH-specific IL-17-producing T cells were not correlated with BP180-specific IL-5-producing Th2 cells in BP patients in contrast to EP with pruritic disorders (**Figure 3**), indicating a diverging role of these cells in both disorders. Although Th17 cells at site of the dermal-epidermal detachment in BP patients have been reported (43, 47, 48), IL-17 may also be secreted by innate immune cells including macrophages, neutrophils, and dendritic cells (49–51).

Another finding of our study is the significant suppression of autoreactive T cells in patients with BP on topical immunosuppressive treatment (**Figure 4** **).** Topical super potent glucocorticoids (GC) as monotherapy are currently considered the first line treatment in moderate and severe BP (52, 53) although other therapeutic options are also discussed (54). In our study, clobetasol propionate ointment was topically applied in 16 BP patients at an initial dose of approximately 40 g daily for 1 month, and then was slowly tapered over the following 12 months. Previous studies had already shown that topical GC are absorbed systemically in amounts sufficient to suppress the hypothalamus-pituitary-adrenal axis in a vast number of patients (55–60), leading to the development of Cushing syndrome (57, 61). Accordingly, we observed BP180-specific autoreactive Th1-, Th2-, and Th17 cells to be significantly suppressed in a subgroup of BP patients treated with clobetasol propionate ointment compared to naïve BP patients who did not receive systemic or topical GC treatment (**Figure 4** **).** Topical GC has been reported as effective also in reducing pruritus through their anti-inflammatory properties (62–65). Furthermore, it has been reported that topical application of GC can reduce pruritus affecting the pathway of histamine-induced itch (66, 67). GC have immunosuppressive effects on pro-inflammatory T cells, inhibiting synthesis and function of Th17 and Th1 effector cytokines, such as IL-17 and IFN-γ (66).

In conclusion, the results of this pilot study show Th1 cell recognition of BP180 in individual EP with pruritic disorders which differs from preferential autoreactive Th2 responses in patients with BP and nBP. Autoreactive Th17 responses were only seen in a few EP with pruritic disorders and patients with BP. Further investigations need to elucidate the precise role of autoreactive Th1, Th2, and Th17 responses in the induction and perpetuation of BP and related pruritic disorders. Moreover, our findings provide further insights into systemic immunosuppressive effects of topical GC treatment in BP.

## Data Availability Statement

The original contributions presented in the study are included in the article/**Supplementary Material**. Further inquiries can be directed to the corresponding author.

## Ethics Statement

The studies involving human participants were reviewed and approved by Ethik-Kommission des Fachbereich Medizin Marburg Baldingerstraße/Postfach 2360, 35032, Marburg. The patients/participants provided their written informed consent to participate in this study. Written informed consent was obtained from the individual(s) for the publication of any potentially identifiable images or data included in this article.

## Author Contributions

MF and VK performed the experiments. LS, DD, JP, and RP analyzed the data and wrote the manuscript. RP supervised the study. FS, DD, HJ, MG, SM, and LH recruited patients. TS, CS, DD, RE, and MH conceived the study and revised the manuscript. All authors contributed to the article and approved the submitted version.

## Funding

This study was supported by grants from the Deutsche Forschungsgemeinschaft (DFG) to MH (He 1602/13-1, 13-2) and CS (SI-1281/5-1) and by FOR 2497 Pegasus to MH, RE, and CS. DD, LS, and FS were supported by a Clinician Scientist Program associated with FOR 2497.

## Conflict of Interest

The authors declare that the research was conducted in the absence of any commercial or financial relationships that could be construed as a potential conflict of interest.

## Dedication

This work is dedicated to Marcel Jonkman, late professor and chairman of the Department of Dermatology, University of Groningen, Netherlands. Over many years, Prof. Jonkman had been one of the international protagonists in bullous pemphigoid research and is one of the fathers of the concept that bullous pemphigoid is a clinically heterogeneous autoimmune bullous disorder associated with pruritus and age related loss of immune tolerance against components of the basement membrane zone.

## Abbreviations

BMZ, basement membrane zone; BP, bullous pemphigoid; BPDAI, Bullous Pemphigoid Disease Area Index; CPDA, citrate-phosphate-dextrose-adenin; EP, elderly patients; GC, glucocorticoids; HC, healthy controls; IL, interleukin; IFN-γ, interferon-γ; nBP, non-bullous pemphigoid; NC, non-collagenous; PBMC, peripheral blood mononuclear cells; Treg, T regulatory; Th, T helper; TNF-α, tumor necrosis factor-α.
