# Supplementary material for: Autoreactive Peripheral Blood T Helper Cell Responses in Bullous Pemphigoid and Elderly Patients With Pruritic Disorders
Source: Front Immunol. 2021 Mar 25;12:569287. doi: 10.3389/fimmu.2021.569287 (PMC8027500; doi:10.3389/fimmu.2021.569287)
Supplement: Supplementary file 1 [file DataSheet_1.docx]

Supplementary Material

**Autoreactive peripheral blood T helper cell responses in elderly patients with pruritic disorders and bullous pemphigoid**

**Dario Didona, Luca Scarsella, Milad Fehresti, Farzan Solimani, Hazem Juratli, Manuel Göbel, Stefan Mühlenbein, Lily Holiangu, Josquin Pieper, Vera Korff, Thomas Schmidt, Cassian Sitaru, Rüdiger Eming, Michael Hertl, Robert Pollmann^*^**

*** Correspondence:** Robert Pollmann: [r.pollmann@mail.de](mailto:r.pollmann@mail.de)

# Supplementary Figures


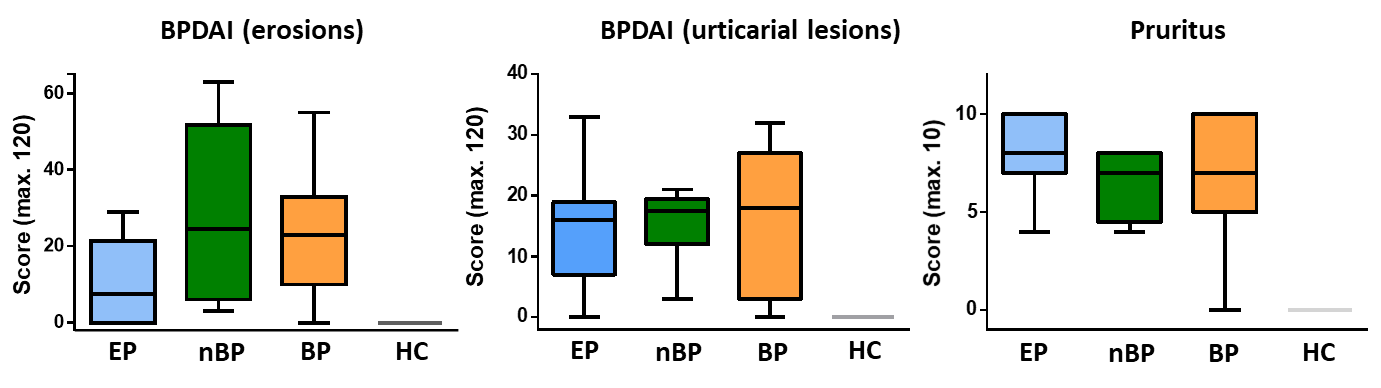


**Supplementary Figure 1:** **Bullous Pemphigoid Disease Area Index (BPDAI) and pruritus assessment for elderly patients with pruritic disorders (EP), non-bullous BP, bullous pemphigoid (BP) and healthy controls (HC).**

# Supplementary Tables

**Supplementary Table 1: IgG autoantibodies against bullous pemphigoid (BP) 180 and BP230 in elderly patients with pruritic disorders, non-bullous pemphigoid (nBP), BP patients and healthy individuals**

| Groups | α-BP180-NH2 -IgG | α-BP230-IgG |
| --- | --- | --- |
|  | p value | p value |
| EP^1^ vs. nBP^2^ | **0.0010** | 0.1546 |
| EP vs. BP^3^ | **0.0002** | **0.0203** |
| EP vs. HC | 0.9613 | 0.4764 |
| nBP vs. BP | 0.2686 | 0.8573 |
| nBP vs. HC | **0.0159** | 0.1984 |
| BP vs. HC | **0.0220** | **0.0461** |

^1^EP, elderly patients with pruritic disorders

^2^nBP, non-bullous pemphigoid

^3^BP, bullous pemphigoid

**Supplementary Table 2: Peripheral blood T cell responses against bullous pemphigoid (BP) 180 in elderly patients with pruritic disorders (EP), non-bullous pemphigoid (nBP), bullous BP and healthy controls (HC)**

| T cell response | Groups | BP180-NH2 | BP180-COOH |
| --- | --- | --- | --- |
|  |  | p value | p value |
| IFN-γ | EP^1^ vs nBP^2^ | 0.5814 | 0.6711 |
|  | EP vs BP | 0.1568 | **0.0123** |
|  | EP vs HC | 0.7055 | 0.2137 |
|  | nBP vs BP^3^ | 0.3199 | **0.0229** |
|  | nBP vs HC | 0.4172 | 0.1271 |
|  | BP vs HC | **0.0249** | **0.0156** |
| IL-5 | EP vs nBP | 0.0638 | 0.7440 |
|  | EP vs BP | **0.0193** | **0.0192** |
|  | EP vs HC | 0.2987 | 0.3316 |
|  | nBP vs BP | **0.0024** | 0.0856 |
|  | nBP vs HC | 0.2642 | 0.7990 |
|  | BP vs HC | **0.0009** | **0.0497** |
| IL-17 | EP vs nBP | 0.4114 | 0.6800 |
|  | EP vs BP | 0.0922 | **0.0120** |
|  | EP vs HC | 0.4651 | 0.2345 |
|  | nBP vs BP | 0.6654 | 0.1907 |
|  | nBP vs HC | 0.1344 | 0.4017 |
|  | BP vs HC | **0.0016** | 0.0629 |

^1^EP, elderly patients with pruritic disorders

^2^nBP, non-bullous pemphigoid

^3^BP, bullous pemphigoid

**Supplementary Table 3: Impact of treatment with topical glucocorticoids (clobetasol propionate ointment) on peripheral blood T cell responses against pemphigoid (BP) 180 in BP patients**

| T cell response | BP^1^  group | BP180-NH2 | BP180-COOH |
| --- | --- | --- | --- |
|  |  | p value | p value |
| IFN-γ | untreated vs treated | **0.0086** | **0.0408** |
| IL-5 | untreated vs treated | **0.0026** | 0.0815 |
| IL-17 | untreated vs treated | **0.0010** | **0.0055** |

^1^BP, bullous pemphigoid
